# TMC5 is Highly Expressed in Human Cancers and Corelates to Prognosis and Immune Cell Infiltration: A Comprehensive Bioinformatics Analysis

**DOI:** 10.3389/fmolb.2021.810864

**Published:** 2022-01-13

**Authors:** Hui Zhang, Xu Zhang, Weiguo Xu, Jian Wang

**Affiliations:** Department of General Surgery, Jiangsu Cancer Hospital, Jiangsu Institute of Cancer Research, The Affiliated Cancer Hospital of Nanjing Medical University, Nanjing, China

**Keywords:** TMC5, cancer prognosis, immune cell infiltration, cancer pathways, drug response

## Abstract

**Background:** The oncological role of TMC5 in human cancers has only been revealed partially. We performed integrated bioinformatics analysis to provide a thorough and detailed insight of associations between TMC5 and tumorigenesis, cancer progression, and prognosis.

**Methods:** With reference to the accessible online databases, the TMC5 expressions in tumor tissues and corresponding normal tissues, different pathological stages, and various cancer cells were analyzed, while the protein levels of TMC5 in different cancers were also inspected. Meanwhile, the prognostic value of TMC5 expression in multiple cancers as well as in advanced-stage patients was investigated. Furthermore, the mutational data of TMC5 and its correlation with cancer prognosis were assessed. Moreover, the association between the TMC5 level and immune cell infiltration was evaluated. Next, TMC5-related pathway alterations and drug responses were summarized. Finally, the TMC5 based protein network was generated, and relevant enrichment was performed.

**Results:** In our study, the expression level of TMC5 was significantly higher in the tumor tissue than that of the normal tissues in most cancer types. Fluctuations of TMC5 levels were also observed among different pathological stages. In the meantime, the protein level elevated in the tumor tissue in the cancers enrolled. Moreover, the expression of TMC5 was not only prognostic for overall survival (OS) or recurrence free survival (RFS) in various types of cancers but also correlated to OS in patients with more advanced cancers. Additionally, the mutational status of TMC5 is also associated with prognosis in cancer patients. It is worth noting that the TMC5 level was closely related to immune cell infiltrations, especially in ESCA, TGCT, and USC. The TMC5 expression was also identified as an activator for pathways including PI3K/AKT, RAS/MAPK, and TSC/mTOR, proved to be associated with multiple drug responses and assessed to be interactive with the TMEM family.

**Conclusion:** TMC5 might function as a potential marker for cancer survival and immune responses.

## Introduction

The transmembrane channel (TMC)–like family includes 8 members (TMC1 to TMC8). So far, various studies demonstrated a close relation between the TMC family and human cancers. For instance, TMC6 and TMC8 mutations were not only reported to contribute to cervical cancer susceptibility (Castro et al., 2012) but also correlated to increased risk of skin cancer (Madeleine et al., 2014). In the meantime, TMC7 was identified as a potential prognostic biomarker for pancreatic cancer (Cheng et al., 2019); the same association was discovered between TMC8 and hepatocellular carcinoma (HCC) (Lu et al., 2017), TMC4, and breast cancer (Aushev et al., 2019), TMC3 and colorectal cancer (CRC) (Zhou et al., 2020), TMC8 and head and neck squamous cancer (Lin et al., 2021). As a member of the TMC family, TMC5 was also detected to be related to human cancers. TMC5 was found expressed especially in chromophobe renal cell carcinoma (RCC) (Yusenko and Kovacs, 2009). Meanwhile, overexpression of TMC5 was observed in intrahepatic cholangiocarcinoma (ICC) (Subrungruanga et al., 2013) and prostate cancer (PC) (Zhang et al., 2019). TMC5 is also correlated with tumor microenvironments in HCC and associated with prognosis in HCC and lung adenocarcinoma (LUAD) (Arroyo et al., 2020; Pan et al., 2020). However, whether the oncologic role of TMC5 was limited to certain cancers or broadly applicable is still not clear; therefore, we carried out a bioinformatics analysis to offer a comprehensive view.

## Methods

### Data Acquisition and Processing

#### TCGA

We downloaded cancer-related data of the 33 tumor patients in The Cancer Genome Atlas (TCGA) database, including tumor RNA-seq data (TCGA) (https://portal.gdc.cancer.gov/), TMB (tumor mutation burden), MSI (microsatellite instability), and clinical information. TMB and MSI were derived from the article published earlier (Bonneville et al., 2017; Thorsson et al., 2018).

#### TIMER2

We used the TIMER2 (tumor immune estimation resource, version 2, http://timer.comp-genomics.org) (Li et al., 2020) to assess the expression of TMC5 in both tumor and adjacent normal tissues in different cancers. By inputting “TMC5” in the “Gene_DE” query box, we were able to obtain the specific expression situations in various cancers from TCGA database. (The full name of the tumors analyzed and their corresponding abbreviations are provided in Table 1.)

**TABLE 1:** Full name of the tumors analyzed and their corresponding abbreviations.

Abbreviations	Full name
ACC	Adrenocortical carcinoma
BLCA	Bladder urothelial carcinoma
BRCA	Breast invasive carcinoma
CESC	Cervical squamous cell carcinoma and endocervical adenocarcinoma
CHOL	Cholangiocarcinoma
COAD	Colon adenocarcinoma
DLBC	Lymphoid neoplasm diffuse large B-cell lymphoma
ESCA	Esophageal carcinoma
GBM	Glioblastoma multiforme
HNSC	Head and neck squamous cell carcinoma
KICH	Kidney chromophobe
KIRC	Kidney renal clear cell carcinoma
KIRP	Kidney renal papillary cell carcinoma
LAML	Acute myeloid leukemia
LGG	Brain lower grade glioma
LIHC	Liver hepatocellular carcinoma
LUAD	Lung adenocarcinoma
LUSC	Lung squamous cell carcinoma
MESO	Mesothelioma
OV	Ovarian serous cystadenocarcinoma
PAAD	Pancreatic adenocarcinoma
PCPG	Pheochromocytoma and paraganglioma
PRAD	Prostate adenocarcinoma
READ	Rectum adenocarcinoma
SARC	Sarcoma
SKCM	Skin cutaneous melanoma
STAD	Stomach adenocarcinoma
TGCT	Testicular germ cell tumors
THCA	Thyroid carcinoma
THYM	Thymoma
UCEC	Uterine corpus endometrial carcinoma
UCS	Uterine carcinosarcoma
UVM	Uveal melanoma

#### GEPIA

For certain kinds of cancers lacking the data of adjacent normal tissue in TCGA, we used corresponding data from the Genotype-Tissue Expression (GTEx) database and visualize the data through Gene Expression Profiling Interactive Analysis (GEPIA, http://gepia.cancer-pku.cn) (Tang et al., 2019), using the box plot module. The detailed settings were “*p*-value cutoff = 0.01, log2FC (fold change) cutoff = 1,” and “Match TCGA normal and GTEx data.” We also explored the expression of TMC5 among different pathological tumor stages via the “Pathological Stage Plot” of GEPIA. The expression level was calculated as log2 (transcripts per million (TPM) +1). Under the “Similar Genes Detection” module, we explored 100 genes similar to TMC5 based on TCGA cancer data.

#### UALCAN

We used the UALCAN portal (http://ualcan.path.uab.edu) (Chandrashekar et al., 2017). Using the subcategory CPTAC (Chen et al., 2019) and selecting “TMC5” as the targeted gene, we were able to explore the proteomic level of TMC5 in BRCA, COAD, LUAD, and UCEC.

### The Human Protein Atlas

The expression results in tumor tissues for TMC5 were obtained from the Human Protein Atlas (https://www.proteinatlas.org) (Uhlen et al., 2015). By inputting the targeted gene symbol ids, we were able to compare the immunohistochemistry stain results among individuals’ tumor samples and the survival outcomes in different expression groups of late-stage patients.

#### CCLE

We accessed the Cancer Cell Line Encyclopedia (CCLE, https://sites.broadinstitute.org/ccle/) website (Nusinow et al., 2020) and obtained the RNA-seq expression differential data of human cancer cell lines by restraining the search range within “Homo Sapien,” “TMC5,” and “Expression 21Q2 Public.” The expression distribution of TMC5 was further analyzed using log2 (TPM+1) as the x-axis using the depmap tool (https://depmap.org/portal/interactive/).

#### KM Plotter

We used the Kaplan–Meier Plotter (http://kmplot.com/analysis/) (Gyorffy, 2021) to analyze the OS and RFS between TMC5 high and low groups across various types of cancers. By selecting the mRNA RNA-seq module (Nagy et al., 2021), we got access to the pan-cancer analysis. After inputting “TMC5” in the Gene symbol box, choosing between the OS/RFS and the corresponding cancer type, we generated the survival plot.

#### cBioPortal

The website of cBioPortal (https://www.cbioportal.org/) offers the overview and detailed mutation-related information of TMC5. For the “Quick select” section, we selected the “TCGA Pan Cancer Atlas Studies” and further entered “TMC5” in the query box for genetic characteristics. The retrieved data consisted of the following aspects: 1) “Cancer Types Summary” module, which demonstrated the mutation type, copy number alteration (CNV), and their distributions across TCGA cancers; 2) three-dimensional display of the structure of the TMC5 transcript; 3) the mutated site information of TMC5; and 4) comparison of the OS and disease-specific survival (DSS) across TCGA cancers with or without genetically altered TMC5.

#### GCSA

We also obtained the methylation level of TMC5 provided by the GCSA website (Gene Set Cancer Analysis, Guo Lab, College of Life Science and Technology, HUST, China, http://bioinfo.life.edu.cn/GSCA/#/) (Liu et al., 2018). By selecting the mutation label and checkboxes of “Differential methylation” and “Methylation & Survival,” we were able to achieve visualized information about the methylation level of TMC5 in different cancers and its relation with survival. We demonstrated the correlation between OS/progression-free survival (PFS)/DSS and TMC5 methylation level in KIRC. Accordingly, we analyzed the pathways that might be potentially related to TMC5 in the “Expression” module. We got access to the correlations between TMC5 expression and sensitivities of anticancer drugs from databases of Genomics of Drug Sensitivity in Cancer (*GDSC*) and the Cancer Therapeutics Response Portal (*CTRP*) through GCSA and demonstrated the top 10 correlated drugs in the radar plot.

#### TISIBD

We used the TISIBD (Ru et al., 2019), an integrated repository portal for tumor-immune system interactions website, to explore the correlation between TMC5 and immune cell infiltration. By inputting “TMC5” in the query box and further selecting the corresponding gene symbol, we obtained the heatmaps of TMC5 expression and lymphocytes, immunomodulators, and chemokines infiltration levels. We also observed the most correlated immune cell and cancer types by plotting the scattering diagram.

#### STRING

To investigate the interaction network involving TMC5, we used the STRING tool (https://string-db.org/), to obtain a comprehensive network of TMC5-binding proteins. We first inputted “TMC5” and “Homo sapiens,” respectively, for protein name and organism in the query box. We set the confidence (0.150), most protein number (50), and determined the sources from text mining, experiments, databases, co-expression, neighborhood, gene fusion, and co-occurrence.

#### DAVID

We then uploaded the protein list combining the results from GEPIA and STRING to Database for annotation, visualization, and integrated discovery (DAVID, https://david.ncifcrf.gov/tools.jsp) (Huang et al., 2009a; Huang et al., 2009b) and chose the selected identifier to be “OFFICIAL_GENE_SYMBOL” and species to be “Homo sapiens.” The data of the functional annotation were further analyzed and visualized by the “tidyr” and “ggplot2” R packages. We also conducted and visualized gene ontology (GO) enrichment based on the involved proteins applying the “clusterProfiler” R package.

### Statistical Analysis

Data processing was based on R software (R-4.0.1,64-bit). The R ratio was calculated using the Spearman correlation test. The *p* value was two-sided, and *p* < 0.05 is considered statistically significant.

## Results

### TMC5 Expressed Differentially Between Tumor and Normal Tissues

**FIGURE 1:**
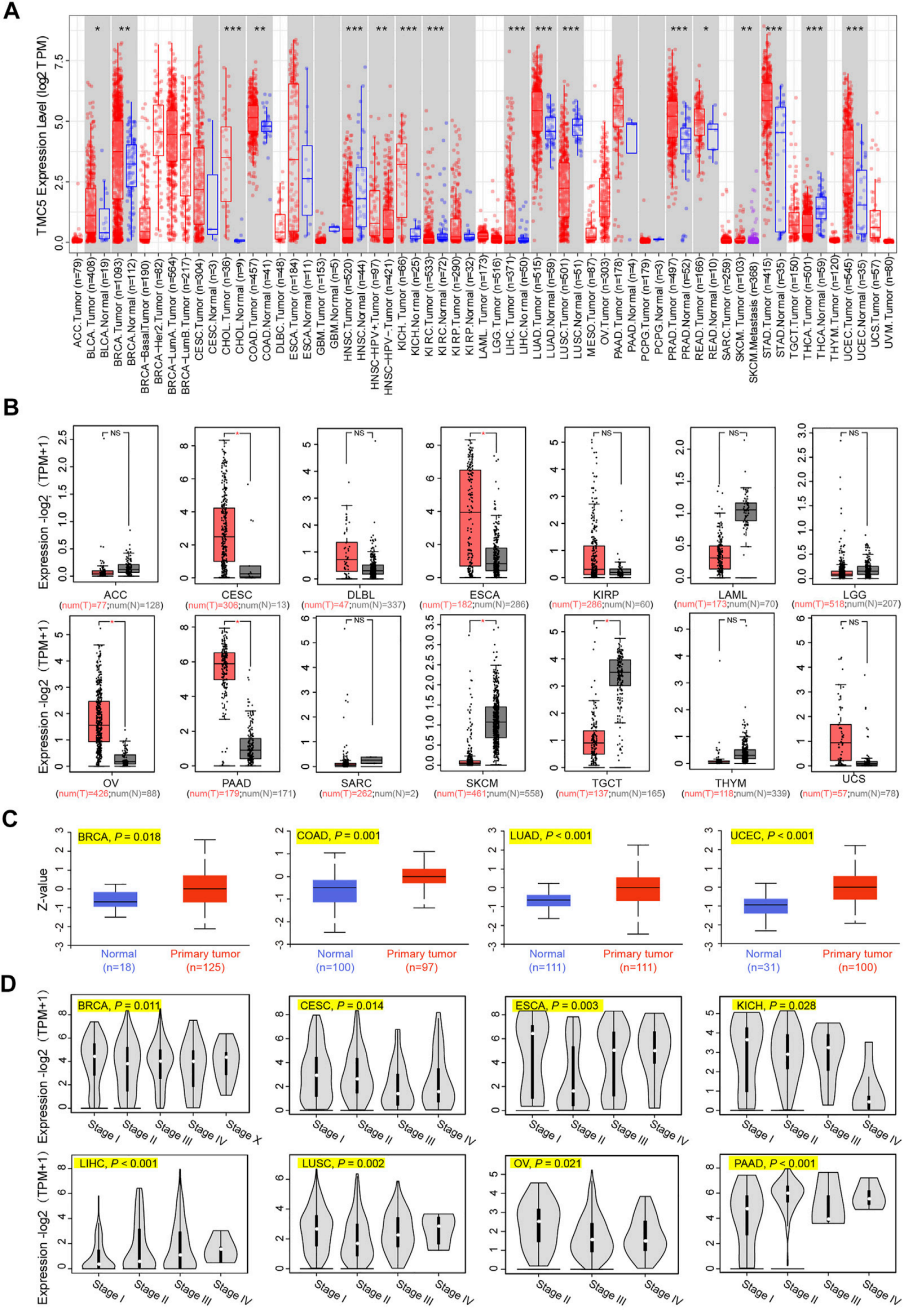
Expression level of TMC5 in different databases. **(A)** Analysis of mRNA expression of TMC5 in different cancers or in cancers of specific subtypes, based on TIMER2, *p < 0.05; **p < 0.01; ***p < 0.001. **(B)** Due to the lack of normal tissue data in TCGA, the corresponding normal tissues of ACC, CESC, DLBC, ESCA, KIRP, LAML, LGG, OV, PAAD, SARC, SKCM, TGCT, THYM, and UCS from the GTEx database were set as control. Comparisons of the TMC5 mRNA expression level in primary tumor (T) and normal tissue (N) are presented in box plot by GEPIA, *p < 0.05. **(C)** Total protein levels of TMC5 in BRCA, COAD, LUAD, and UCEC were analyzed through CPTAC database. **(D)** TCGA based mRNA expression of TMC5 in different pathological stages of various cancers (stage I, stage II, stage III, and stage IV of CESC, ECSA, KICH, LIHC, LUSC, and PAAD; stage I, stage II, stage III, stage IV, and stage X of BRCA; stage II, stage III, and stage IV of OV) were compared using GEPIA, with log2 (TPM+1) adopted for log-scale.

**FIGURE 2:**
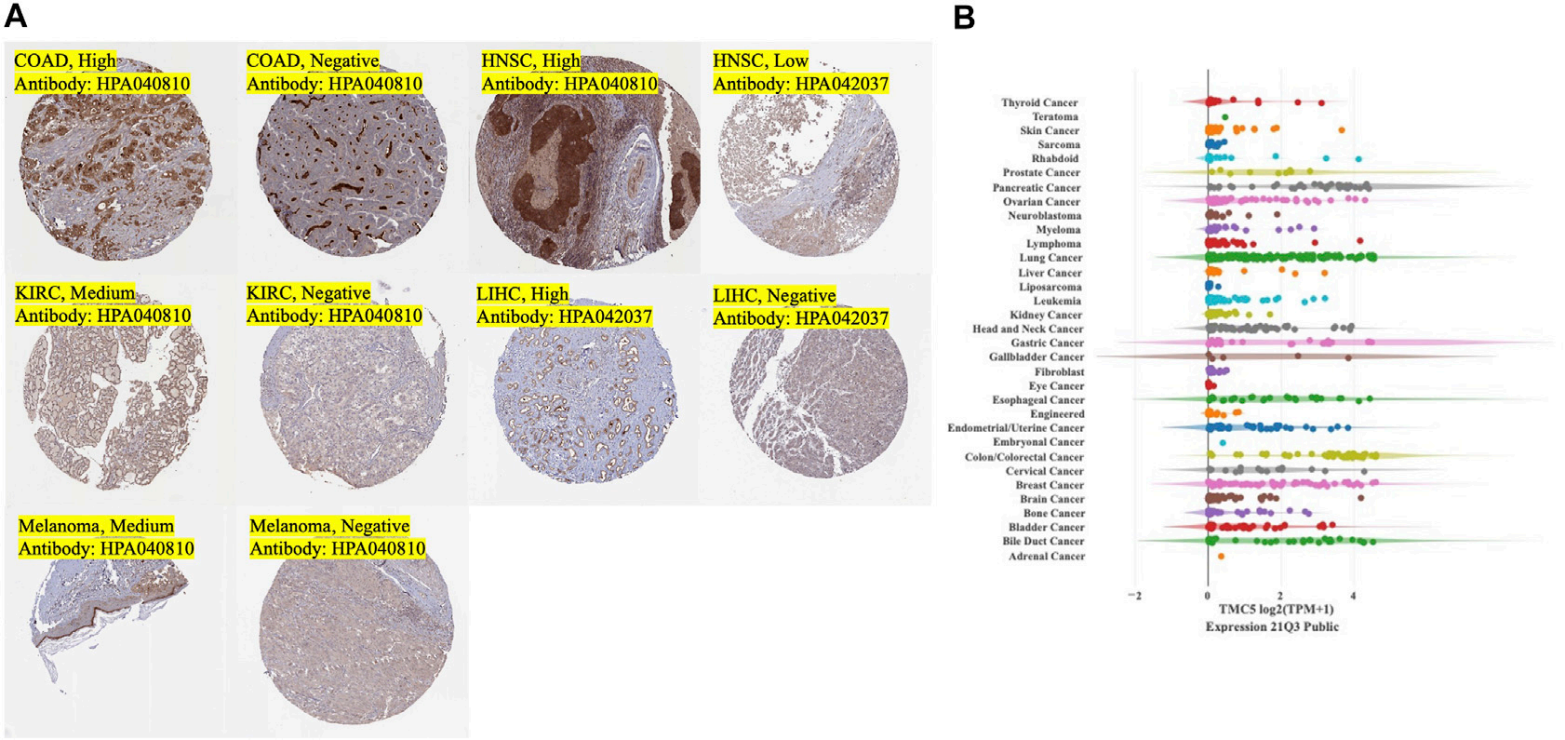
Expression of TMC5 in tumor tissues and expression of TMC5 in human cancer cell lines. **(A)** Typical IHC stain of tumor tissues with medium to strong stain or negative stain. **(B)** Overall summary of TMC5 expressions in human cancer cell lines.

First, we investigated the expression level of TMC5 in 33 human cancers in TCGA cohort with the corresponding normal tissue as control (Figure 1A). TIMER2 showed a broad overexpression of TMC5 in most cancer tissues, including BLCA, BRCA, CHOL, COAD, KICH, LIHC, LUAD, PRAD, READ, STAD, and UCEC (*p < 0.05, **p < 0.01, ***p < 0.001). Meanwhile, low expression of TMC5 in tumor was observed in HNSC, KIRC, LUSC, and THCA (*p < 0.05, **p < 0.01, ***p < 0.001). Additionally, the expression of TMC5 was also found to elevate in HPV-positive HNSC compared to the HPV-negative subgroup (**p < 0.01). On the other hand, GEPIA demonstrated that TMC5 was highly expressed in CESC, ESCA, OV, and PAAD, while lowly expressed in SKCM and TGCT (*p < 0.05, Figure 1B). Consequentially, as shown in Figure 1C, the protein level of TMC5 was assessed to be significantly higher in the primary tumors than in the normal tissues in BRCA (*p* = 0.018), COAD (*p* = 0.001), LUAD (*p* < 0.001) and UCEC (*p* < 0.001). We further evaluated the expression of TMC5 in different pathological stages in human cancers and uncovered a statistically significant correlation between the TMC5 level and cancer stages in BRCA, CESC, ESCA, KICH, LIHC, LUSC, OV, and PAAD, Especially, in LIHC, LUSC, and PAAD, the TMC5 level was positively correlated with cancer stages (Figure 1D). We also studied the expression level of TMC5 in tumor tissues and human cancer cell lines with reference to The Human Protein Atlas project and CCLE, as shown in Figure 2.

### TMC5 Expression Correlated to Cancer Prognosis

**FIGURE 3:**
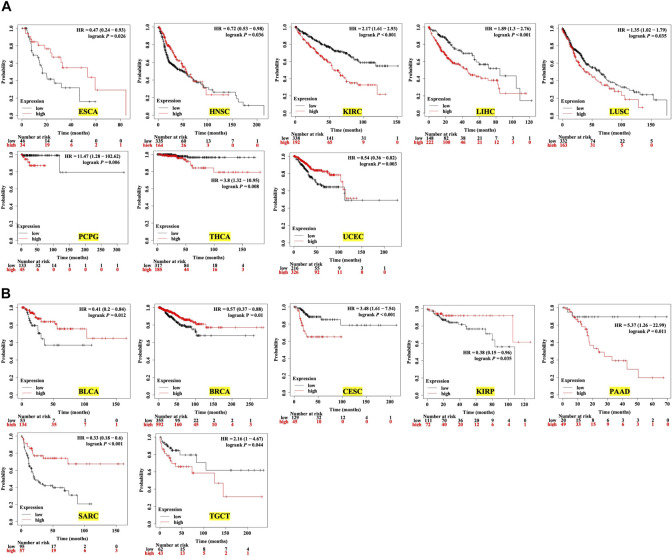
Survival analysis of TMC5 expression in multiple human cancers (HR: hazard ratio). **(A)** Association between TMC5 expression and overall survival in various cancers. High expression of TMC5 was closely correlated to shorter overall survival in KIRC, LIHC, LUSC, PCPG, and THCA; contrarily, high expression of TMC5 also associated with longer OS in ECSA, HNSC, and UCEC. **(B)** TMC5 expression associated with RFS in cancer patients, in BLCA, BRCA, KIRP, and SARC; high TMC5 level associated with longer RFS, while in CESC, PAAD, and TGCT, highly expressed TMC5 correlated to shorter RFS.

Next, we analyzed the prognostic value of the expression level of TMC5 for OS and RFS in different cancers. The results demonstrated that high expression of TMC5 was linked to the poor OS in KIRC (*p* < 0.001), LIHC (*p* < 0.001), LUSC (*p* = 0.035), PCPG (*p* = 0.006), and THCA (*p* = 0.008). Inconsistently, for ESCA (*p* = 0.026), HNSC (*p* = 0.036), and UCEC (*p* = 0.003), lowly expressed TMC5 was associated with shorter OS (Figure 3A). As for RFS, a high level of TMC5 correlated to shorter RFS in CESC (*p* < 0.001), PAAD (*p* = 0.011), and TGCT (*p* = 0.044); however, in BLCA (*p* = 0.012), BRCA (*p* = 0.01), KIRP (*p* = 0.035), and SARC (*p* < 0.001), patients with high TMC5 level had longer RFS (Figure 3B).

**FIGURE 4:**
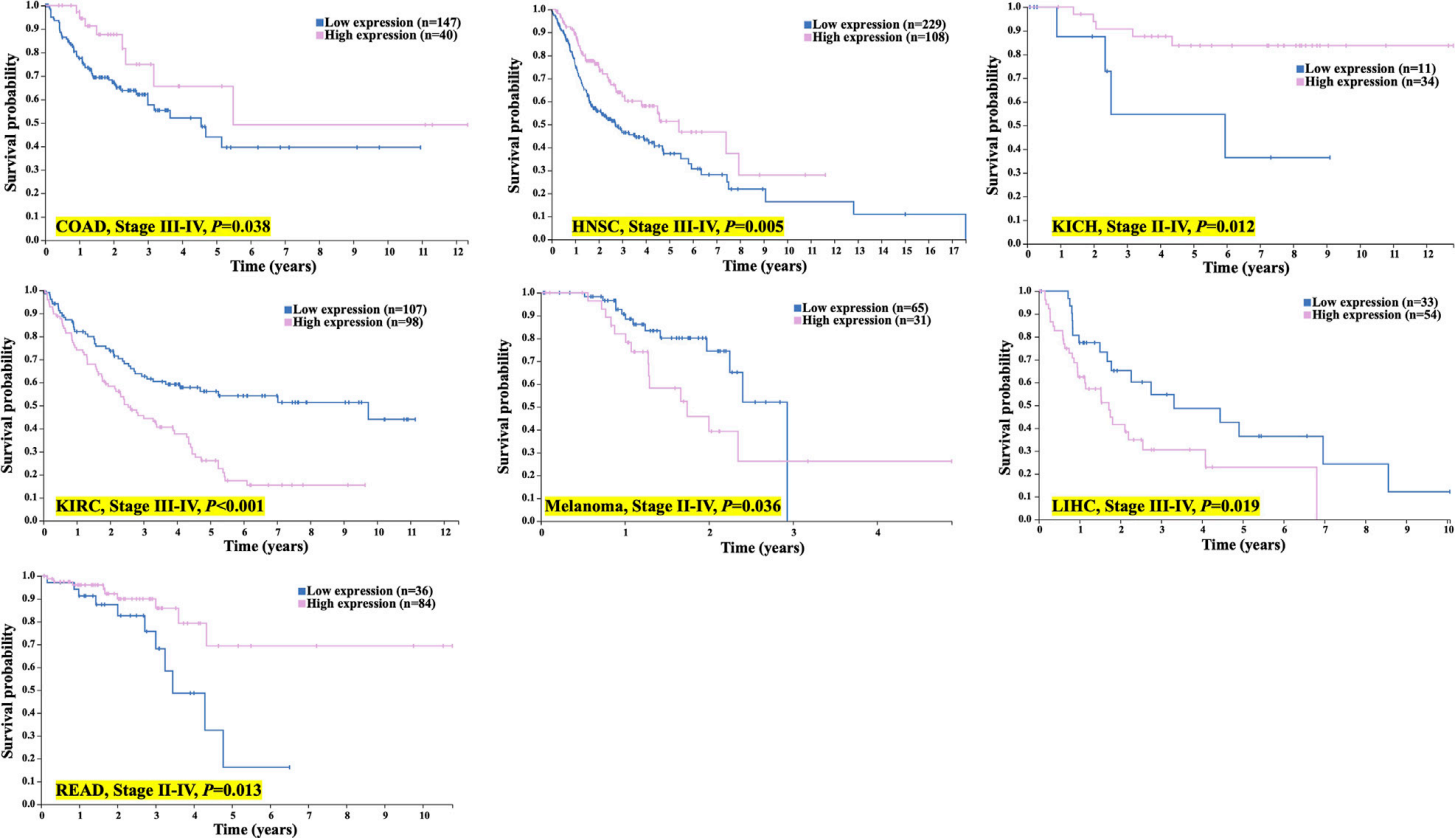
TMC5 expression associated with OS in advanced cancer patients. In COAD, HNSC, KICH, and READ, high TMC5 level associates with longer OS; while in KIRC, melanoma, and LIHC, highly expressed TMC5 correlated to shorter OS.

Moreover, for patients with more advanced tumor stages (II-IV), the expression of TMC5 was also closely associated with OS. In advanced cancers including COAD (stages III-IV, *p* = 0.038), HNSC (stages III-IV, *p* = 0.005), KICH (stages II-IV, *p* = 0.012), and READ (stages II-IV, *p* = 0.013), longer OS was achieved in patients with elevated TMC5 expression, while for KIRC (stages III-IV, *p* < 0.001), melanoma (stages II-IV, *p* = 0.036), and LIHC (stage III-IV, *p* = 0.019), highly expressed TMC5 was correlated to shorter OS (Figure 4).

### Mutational Data

**FIGURE 5:**
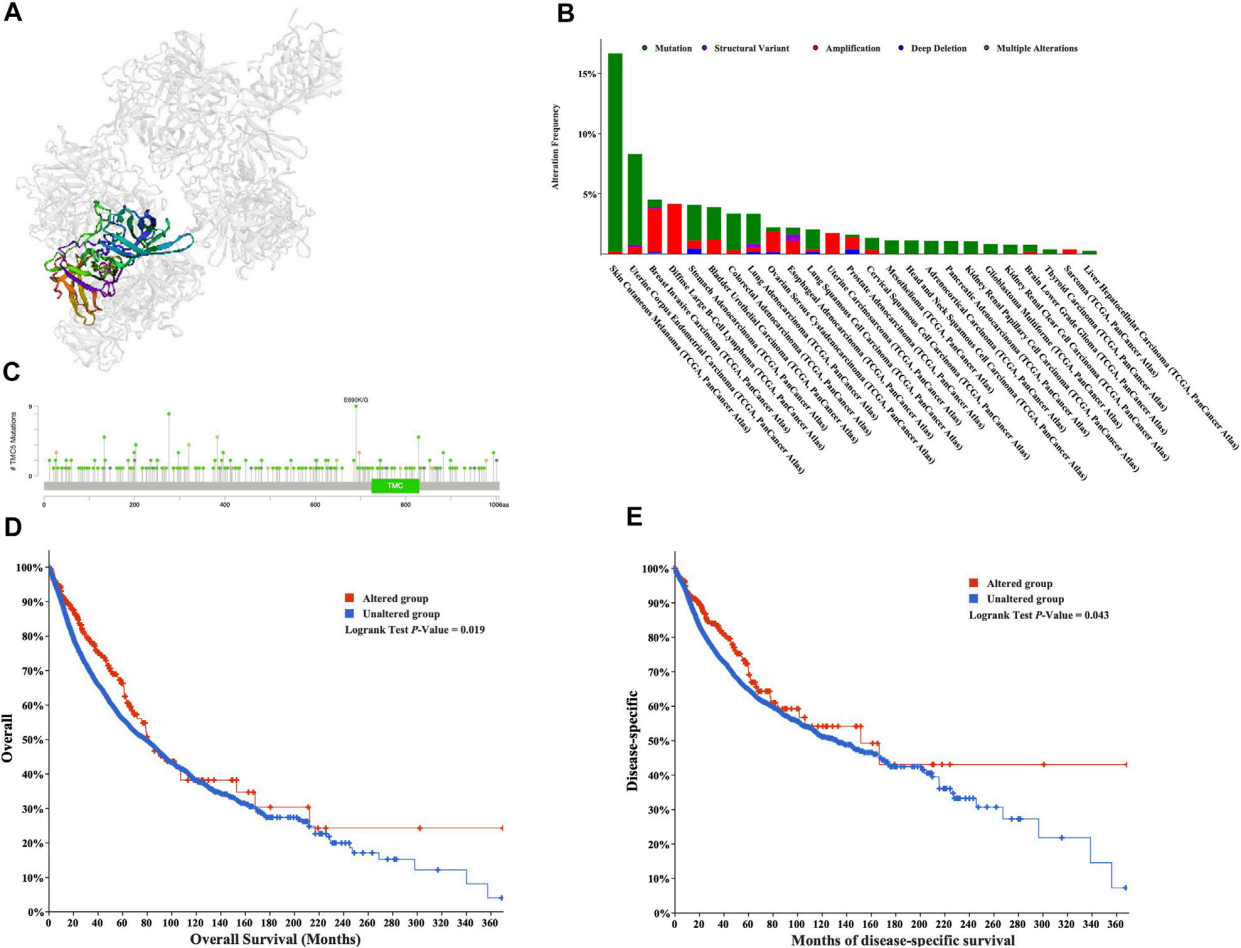
Mutation analysis of TMC5 in different cancers. **(A)** 3D structure of TMC5 gene transcript. **(B)** Summary of mutation types of TMC5 (structural variant data, mutation data, and copy number variant data) and the distribution among different cancers. **(C)** Hot spots of mutation of TMC5. **(D,E)** Alteration of TMC5 was associated with shorter OS and DSS.

We obtained the mutational data of TMC5 from the cBioPortal website. Based on TCGA pan-cancer atlas, a three-dimensional image of the TMC5 transcript was acquired (Figure 5A). In the meantime, SKCM ranked first in TMC5 alteration frequency, followed by UCEC and BRCA (Figure 5B). The distribution of the mutation spot is shown in Figure 5C. Further analysis revealed significant differences in OS (*p* = 0.019) and DSS (*p* = 0.043) between patients with and without TMC5 alterations (Figures 5D,E).

**FIGURE 6:**
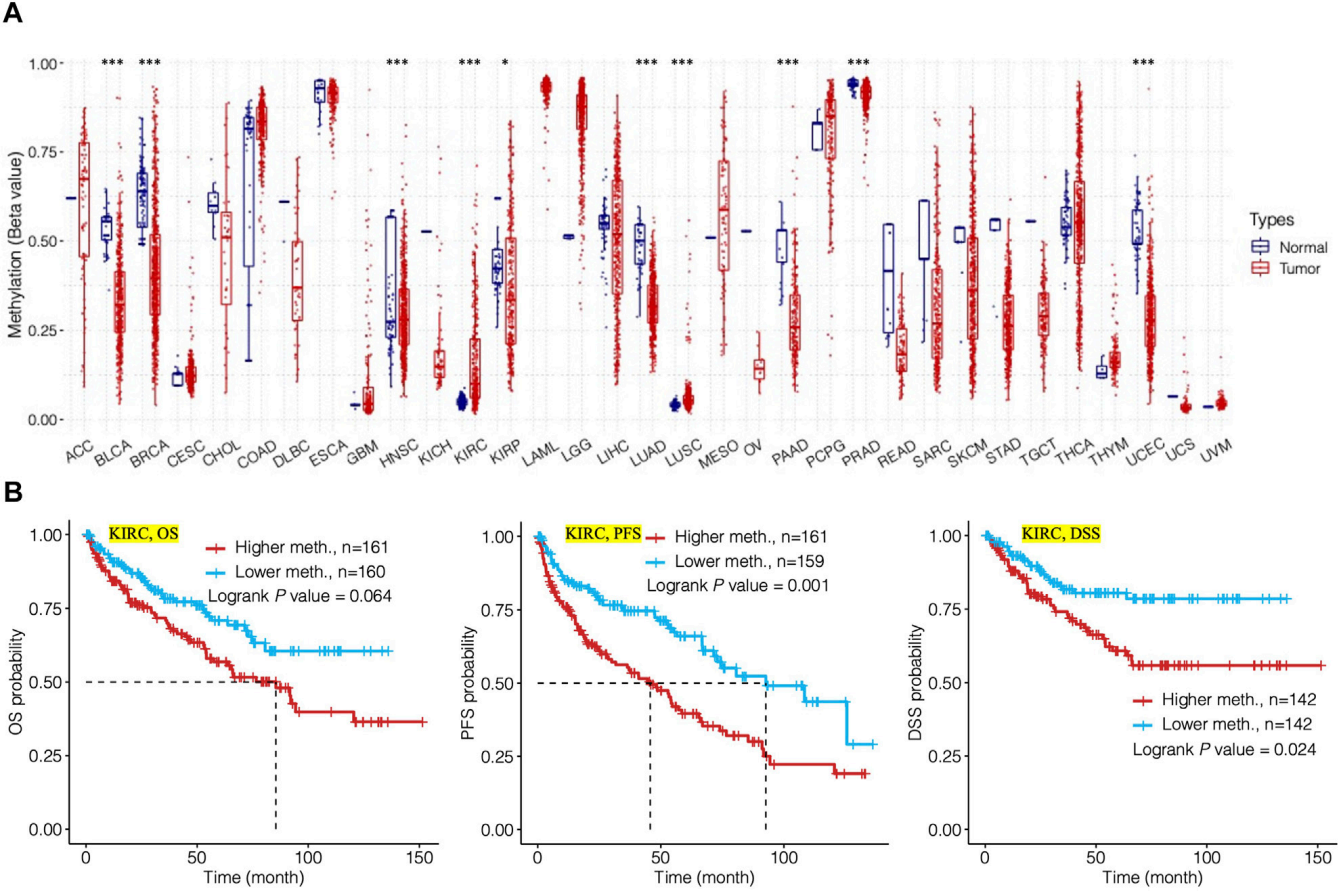
Analysis of methylation level of TMC5 in human cancers and its relation to prognosis. **(A)** Methylation level of TMC5 in different cancers; significant differences were observed in KIRP (*p < 0.05) and BLCA, BRCA, HNSC, KIRC, LUAD, LUSC, PAAD, PRAD, and UCEC (***p < 0.001). **(B)** In KIRC, high methylation level of TMC5 was significantly correlated to shorter PFS and DSS, as well as OS, though not statistically significant.

The methylation level of TMC5 was investigated through the GCSA platform. For cancers including BLCA (*p* < 0.001), BRCA (*p* < 0.001), HNSC (*p* < 0.001), KIRC (*p* < 0.001), KIRP (*p* < 0.05), LUAD (*p* < 0.001), LUSC (*p* < 0.001), PAAD (*p* < 0.001), PRAD (*p* < 0.001), and UCEC (*p* < 0.001), TMC5 was significantly differentially methylated between tumor and normal tissues (Figure 6A). In KIRC (Figure 6B), the methylation level of TMC5 was statistically related to PFS (*p* = 0.001) and DSS (*p* = 0.024), however, was not significantly associated with OS (*p* = 0.064).

### TMC5 Was Involved in Different Protein Networks, Molecular Enrichment, and Pathway Regulation

**FIGURE 7:**
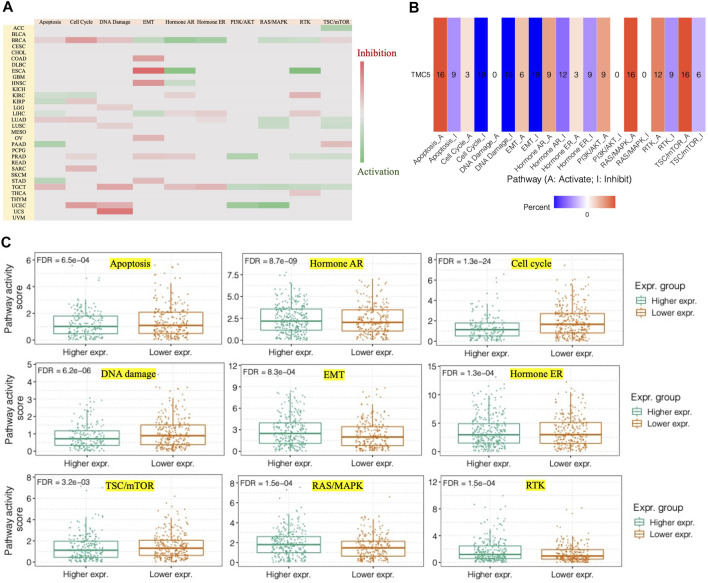
TMC5 expression closely correlated to different pathways. **(A,B)** Heatmaps of associations between high TMC5 level and pathways of apoptosis, cell cycle, DNA damage, EMT, hormone AR, hormone ER, PI3K/AKT, RAS/MAKP, RTK, and TSC/mTOR in various of cancers. **(C)** In BRCA, overexpressed TMC5 acted as an activator for hormone AR, EMT, hormone ER, RAS/MAPK, and RTK, while inhibited apoptosis, cell cycle, and TSC/mTOR.

We continued to analyze the pathways that might be influenced by TMC5 expression using the GCSA. We discovered that TMC5 was in close correlation with various pathways (Figures 7A,B), particularly in BRCA, TMC5 expression activated EMT, hormone AR, hormone ER, RAS/MAPK, and RTK, while inhibited apoptosis, cell cycle, DNA damage, and TSC/mTOR (Figure 7C).

**FIGURE 8:**
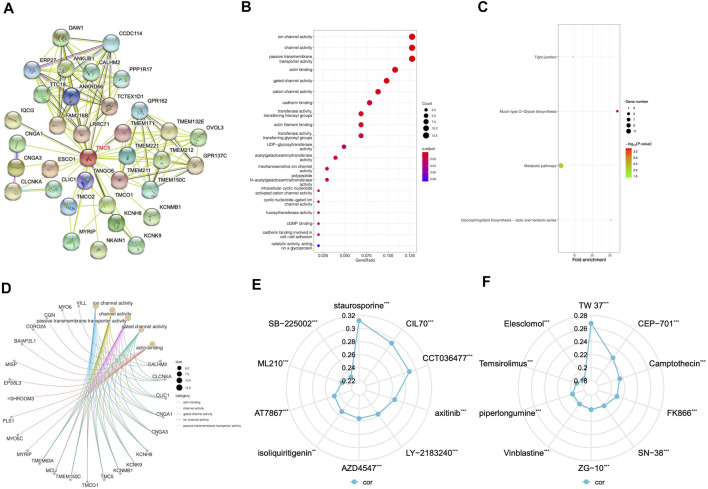
Protein interaction network, KEGG pathway, and GO function analysis and drug response based on TMC5. **(A)** STRING online analysis offered a network formed by possible TMC5 interacting proteins, which was in all consist of 34 proteins. **(B)** KEGG pathway study revealed that these proteins were involved in different channel activities. **(C)** GO analysis emphasized the genes functioned mainly with the metabolic pathways. **(D)** Demonstration of the correlation between the top five functions and the most related protein by GO analysis. **(E,F)** Summary of top 10 agents whose effects were positively related to TMC5 expression based on CTRP and GDSC databases (**p < 0.01, ***p < 0.001).

By using STRING, we generated the protein-to-protein network that included 34 potential interactors with TMC5 (Figure 8A). Then, based on GEPIA, we obtained 100 similar molecules which had no overlap with the previous result (Table 2), and combined the two groups together. The KEGG and GO enrichments of the proteins were performed, respectively. As shown in Figures 8B,C, these molecules were mainly enriched in different channel activities; on the other hand, the functional role of the protein focused mainly on metabolic pathways. Further demonstration of top 5 functional areas of the proteins is presented in Figure 8D.

**TABLE 2:** Similar and interacting genes to TMC5 by GEPIA and STRING.

Gene symbols									
A									
ABHD17C	AC006042.6	ACSL5	AGR2	ANKRD66	ANKUB1	ARHGEF38	ATP2C2	ATP8B1	
B									
B3GNT3	BAIAP2L1	BCL2L15							
C									
C1orf210	C6orf222	C9orf152	CALHM2	CALML4	CAPN8	CASZ1	CATSPERB	CCDC114	CCDC186
CD2AP	CD46	CEACAM6	CGN	CLCNKA	CLIC1	CMPK1	CNGA1	CNGA3	CORO2A
CREB3L1	CTA-363E6.2	CTA-363E6.6	CTD-2196E14.5	CTD-2196E14.8	CTD-2385L22.1	CTSE			
D									
DAW1	DNMBP								
E									
ELF3	EPCAM	EPS8L3	ERN2	ERP27	ESCO1				
F									
FA2H	FAM216B	FAM83E	FRK	FUT2	FUT3				
G									
GALNT3	GALNT4	GALNT7	GCNT3	GFPT1	GMDS	GPR137C	GPR162	GPR35	GPRC5A
GSKIP									
H									
HID1									
I									
ICA1	IQCG								
K									
KCNH8	KCNK9	KCNMB1							
L									
LINC01207	LIPH	LLGL2	LRRC71						
M									
MANSC1	MCU	MISP	MST1R	MUC1	MUC13	MUC3A	MYO5C	MYO6	MYRIP
N									
NKAIN1									
O									
OVOL3									
P									
PCDH1	PDXDC1	PLEKHS1	PLS1	PPAP2C	PPP1R17	PRR15L	PRSS8		
R									
RALGAPA2	RASEF	RBM47	RHPN2	RP11-304L19.1	RP11-304L19.3	RP11-519G16.3	RP11-747H7.3		
S									
SH3RF1	SHROOM3	SLC35A3	SLC37A1	SLC44A4	SMIM22	SPACA4	STK39		
T									
TANGO6	TBC1D30	TC2N	TCTEX1D1	TJP3	TM9SF3	TMC5	TMCO1	TMCO2	TMEM125
TMEM132E	TMEM150C	TMEM171	TMEM211	TMEM212	TMEM221	TMEM45B	TMEM62	TMEM63A	TMEM87B
TNFRSF11A	TSPAN15	TSPAN8	TTC18						
V									
VILL									

### TMC5 Level Associated With Various Drug Response

A positive correlation between TMC5 expression and drug response was revealed in various chemotherapeutic agents. A wide range of targeted agents including inhibitors for EGFR, Bcl-1, FGFR, AKT, and mTOR showed a better curative effect on patients with higher TMC5 level (Figures 8E,F).

### TMC5 Expression Was Correlated to TMB, MSI, and Immune Cell Infiltration

**FIGURE 9:**
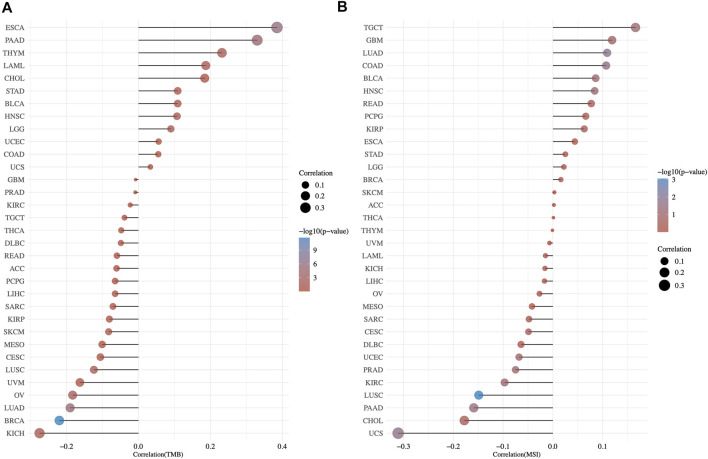
Correlation of TMB/MSI and TMC5 gene expression by spearman analysis. The correlation coefficient between TMC5 and TMB (A)/MSI (B) was presented as the horizontal axis, the ordinate and the size of the dots represented the tumor types and the size of the correlation coefficient, respectively. We used the color of the dots to show the significance of the P value, as the color became redder, the P value got more significant.

With reference to TCGA cohorts, we studied the possible links of TMC5 expression to TMB and MSI (Figure 9). The results showed that in cancers including ESCA (*p* < 0.001, R = 0.385), PAAD (*p* < 0.001, R = 0.33), THYM (*p* < 0.05, R = 0.232), HNSC (*p* < 0.05, R = 0.107), and STAD (*p* < 0.05, R = 0.109), the TMC5 expression level had positive correlation with TMB, while in BRCA (*p* < 0.001, R = −0.22), LUAD (*p* < 0.001, R = −0.19), OV (*p* < 0.01, R = −0.183), LUSC (*p* < 0.01, R = −0.124), and KICH (*p* < 0.05, R = −0.275), the TMC5 level was negatively related to TMB. For MSI, in LUAD (*p* < 0.05, R = 0.109) and COAD (*p* < 0.05, R = 0.107), TMC5 expression showed a positive connection with TMB (*p* < 0.001). Meanwhile, in LUSC (*p* < 0.001, R = −0.149), UCS (*p* < 0.05, R = −0.311) and PAAD (*p* < 0.05, R = −0.159), the TMC5 level was proved to be negatively correlated to MSI.

**FIGURE 10:**
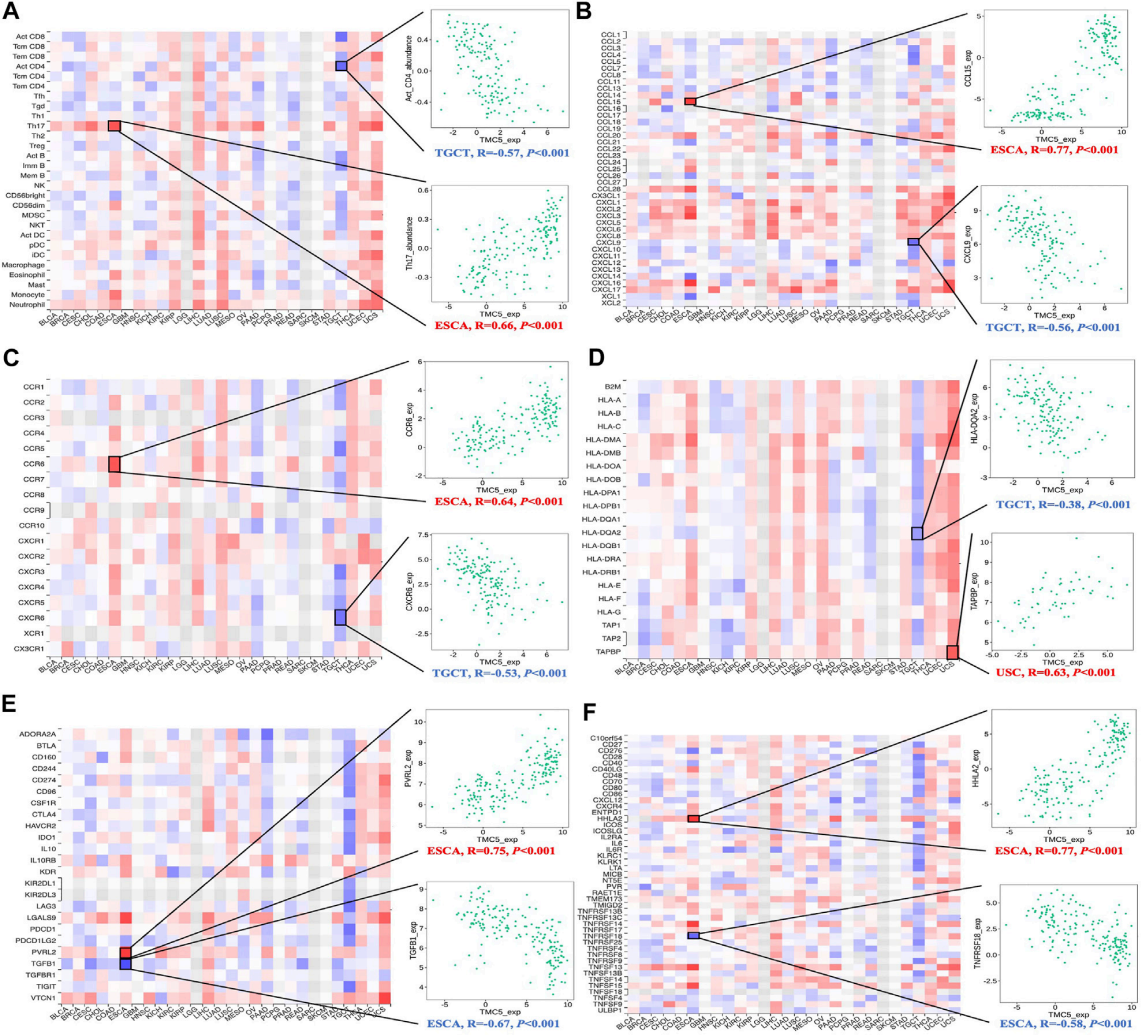
Assessment of correlation between TMC5 expression and immune cell infiltration. Expression of TMC5 was significantly associated with TILs **(A)**, chemokines **(B)**, receptors **(C)**, MHCs **(D)**, immunoinhibitors **(E)** and immunostimulators **(F)**.

At last, we assessed the interrelationship of TMC5 expression and the immune-related molecules. Our results uncovered that TMC5 expression was closely associated with various types of immune cells including immunostimulators, immunoinhibitors, lymphocytes, and chemokines (Figure 10). By further analysis, we discovered statistically meaningful correlations between expressions of TMC5 and Act CD4 (TGCT, R = −0.57, *p* < 0.001), Th17 (ESCA, R = 0.66, *p* < 0.001), CCL15 (ESCA, R = 0.77, *p* < 0.001), CXCL9 (TGCT, R = −0.56, *p* < 0.001), CCR6 (ESCA, R = 0.64, *p* < 0.001), CXCR6 (TGCT, R = −0.53, *p* < 0.001), HLA-DQA2 (TGCT, R = -0.38, *p* < 0.001), TAPBP (USC, R = 0.63, *p* < 0.001), PVRL2 (ESCA, R = 0.75, *p* < 0.001), TGFB1 (ESCA, R = −0.67, *p* < 0.001), HHLA2 (ESCA, R = 0.77, *p* < 0.001), and TNFRSF18 (ESCA, R = −0.58, *p* < 0.001). It is worth noting that, the associations between levels of TMC5 and immune cells in TGCT and ESCA were not limited to some particular cell types but existed in a wide range across the immune molecules.

## Discussion

According to our study, TMC5 expressed differentially in human tumor tissue and normal tissue. Also, its expression level was closely correlated to the OS and cancer stage in many cancers. And, for most of them, high TMC5 level was in close correlation with shorter OS or more advanced stages. Moreover, the alteration status of TMC5 including mutation and methylation was also related to cancer prognosis. These results indicated that TMC5 might be a diagnostic predictor for human cancers. To elaborate the underlying mechanism between TMC5 and carcinogenesis, we studied the pathways that may be regulated by TMC5 expression, and our results showed that high TMC5 level was an activator for PI3K/AKT, RAS/MAPK, and TSC/mTOR. These pathways were reported to be involved in the cell growth, cycle, and death (Fresno Vara et al., 2004; Santarpia et al., 2012); thus, activation of the pathways might result in dysregulation of cell survival and finally develop into tumorigenesis (Porta et al., 2014) and various cancers (Aoki and Fujishita, 2017). So, it is reasonable to speculate that TMC5 also correlates to carcinogenesis and progression.

However, the association between TMC5 expression and RFS or OS in advanced patients was inconsistent. While highly expressed TMC5 is associated with better RFS/OS in some cancer types, the opposite relevance existed in others. It should be stated that cancer patients often receive comprehensive therapy, especially the late-stage population. These therapies might have effects on different targets, resulting in discordant outcomes. Addressing the phenomena, we explored the proteins that interacted with TMC5 and discovered that part of the TMEM family was involved within the network. The TMEM family, which was important in cell membranes (Schmit and Michiels, 2018), also played critical roles in both suppressing and promoting carcinogenesis (Kannan et al., 2001; Atalay et al., 2002; Carles et al., 2006; Doolan et al., 2009; Flamant et al., 2012; Li et al., 2015; Gao et al., 2017; Wang et al., 2017). Due to the complicated and contradictory effects of the TMEM family, TMC5 might also function intricately in human cancers. On the other hand, a high level of TMC5 was assessed to be connected to the drug responses of many chemotherapeutic agents. AT7867, an inhibitor for AKT, and temsirolimus, which suppressed the mTOR, were significantly included due to the close relation between TMC5 expression and PI3K/AKT and TSC/mTOR pathways. Moreover, the effects of various agents targeting other spots or pathways also correlated to TMC5 expression, which stated the function and connection of TMC5 in human cancers might be more complicated and worth further mining.

Considering the inoperable condition in the advanced cancer population, chemotherapy and immunotherapy might be the limited modalities; therefore, the association between the TMC5 level and OS in these patients indicated that the TMC5 level might also serve as a potential marker for treatment response in chemotherapy and immunotherapy. We then detected that TMC5 was correlated to both TMB and MSI in various types of cancers. As broadly accepted markers for immunotherapy, TMB and MSI performed surpassingly in recent studies (Aparicio et al., 2013; Chan et al., 2019; Choucair et al., 2020; van Velzen et al., 2020). The close correlation to TMB and MSI highlighted the potential relevance of TMC5 to immunotherapy. Further research revealed an obvious connection between TMC5 expression and immune cell infiltration. The association was observed broadly in various cancers. Moreover, in TGCT, the TMC5 level was negatively associated with most immunoinhibitors, immunostimulators, MHCs, receptors, and TILs, while in ESCA, the contrary connection was uncovered.

## Conclusion

TMC5 is a potential prognostic and immune marker for human cancers.

## Data Availability

The original contributions presented in the study are included in the article/supplementary material; further inquiries can be directed to the corresponding author.
